# Brief comments on three existing approaches for managing neonates at risk of early-onset sepsis

**DOI:** 10.1186/s13052-021-01107-3

**Published:** 2021-07-18

**Authors:** E. Vaccina, A. Luglio, M. Ceccoli, M. Lecis, F. Leone, T. Zini, G. Toni, L. Lugli, L. Lucaccioni, L. Iughetti, A. Berardi

**Affiliations:** 1 Post Graduate School of Paediatrics, Department of Medical and Surgical Sciences of the Mothers, Children and Adults, University of Modena and Reggio Emilia, via del Pozzo 71, 41124 Modena, Italy; 2 Neonatal Intensive Care Unit, Department of Medical and Surgical Sciences of the Mothers, Children and Adults, University of Modena and Reggio Emilia, via del Pozzo 71, 41124 Modena, Italy; 3 Pediatric Unit, Department of Medical and Surgical Sciences of the Mothers, Children and Adults, University of Modena and Reggio Emilia, via del Pozzo 71, 41124 Modena, Italy

**Keywords:** Newborn, Early-onset sepsis, Group B streptococcus, Prevention, Serial clinical observation, Neonatal Sepsis calculator, Intrapartum antibiotic prophylaxis

## Abstract

**Background:**

Growing concerns regarding the adverse effects of antibiotics during the first days of life and the marked reduction in the incidence of early-onset sepsis (EOS) are changing the clinical practice for managing neonates at risk of EOS. Strategies avoiding unnecessary antibiotics while promoting mother-infant bonding and breastfeeding deserve to be considered.

**Main body:**

We compare strategies for managing newborns at risk of EOS recommended by the American Academy of Pediatrics, which are among the most followed recommendations worldwide. Currently three different approaches are suggested in asymptomatic full-term or late preterm neonates: *i)* the conventional management, based on standard perinatal risk factors for EOS alone, *ii)* the neonatal sepsis calculator, a multivariate risk assessment based on individualized, quantitative risk estimates (relying on maternal risk factors for EOS) combined with physical examination findings at birth and in the following hours and *iii)* an approach entirely based on newborn clinical condition (serial clinical observation) during the first 48 h of life.

We discuss advantages and limitations of these approaches, by analyzing studies supporting each strategy. Approximately 40% of infants who develop EOS cannot be identified on the basis of maternal RFs or laboratory tests, therefore close monitoring of the asymptomatic but at-risk infant remains crucial. A key question is to know what proportion of babies with mild, unspecific symptoms at birth can be managed safely without giving antibiotics.

**Conclusions:**

Both neonatal sepsis calculator and serial clinical observation may miss cases of EOS, and clinical vigilance for all neonates is essential There is a need to assess which symptoms at birth are more predictive of EOS, and therefore require immediate interventions, or symptoms that can be carefully reevaluated without necessarily treat immediately the neonate with antibiotics. Studies comparing strategies for managing neonates are recommended.

## Background

Early onset sepsis (EOS) is sepsis presenting from 0 to 72 h of life [1, 2]. Although its current incidence is lower than in the past [3], EOS is still a problem. Non-specific clinical presentation and low predictive values of common biomarkers complicate the early diagnosis [2], that is essential to prevent life threatening complications. Antibiotics are often given in suspected EOS, but unnecessary antibiotics have potential adverse consequences [4, 5]. The approach for managing neonates at risk of EOS is controversial [1].

## Main body

### Conventional management (CM)

Standard perinatal RFs alone are insufficient for ascertainment of neonatal EOS. Indeed, current diagnostic tests are poorly predictive of EOS in asymptomatic neonates, resulting in unnecessary evaluations and antibiotic exposure for numerous uninfected neonates [2]. In fact, up to 15% of infants are evaluated for EOS, and approximately half of them receive empiric antibiotic treatment for rule out EOS [6].

### The neonatal sepsis calculator (NSC)

A multivariate risk assessment has been developed by Kaiser Permanent Northern California (CA, USA). Individualized, quantitative risk estimates (based on maternal RFs) are combined with physical examination findings. According to perinatal information such as gestational age, duration of membrane rupture, highest maternal temperature during labour, GBS colonization status, intrapartum antibiotic prophylaxis (IAP) and clinical conditions in the first 12 h of life (well appearing, equivocal, or clinically ill, Table 1) a sepsis risk score was developed [8].

**Table 1:** Classification of Infant’s Clinical Presentation according to NSC (available at https://neonatalsepsiscalculator.kaiserpermanente.org/) [7]

Clinical Exam	Description
Clinical Illness	1. Persistent need for NCPAP / HFNC / mechanical ventilation (outside of the delivery room) 2. Hemodynamic instability requiring vasoactive drugs 3. Neonatal encephalopathy /Perinatal depression ▪ Seizure ▪ Apgar Score at 5 min < 5 4. Need for supplemental O2 > 2 h to maintain oxygen saturations > 90% (outside of the delivery room)
Equivocal	1. Persistent physiologic abnormality > 4 h ▪ Tachycardia (HR > 160) ▪ Tachypnea (RR > 60) ▪ Temperature instability (> 100.4 °F or < 97.5 °F) ▪ Respiratory distress (grunting, flaring, or retracting) not requiring supplemental O2 2. Two or more physiologic abnormalities lasting for > 2 h ▪ Tachycardia (HR > 160) ▪ Tachypnea (RR > 60) ▪ Temperature instability (> 100.4 °F or < 97.5 °F) ▪ Respiratory distress (grunting, flaring, or retracting) not requiring supplemental O2 Note: abnormality can be intermittent
Well Appearing	No persistent physiologic abnormalities

The RFs based logistic regression model was developed by using a nested case-control study performed at 14 US centers. This large study included data from 608,014 neonates with ≥34 weeks’ gestation born in the period between 1993 and 2007 [9]. Cases of EOS (*n* = 350) were defined as isolation of a pathogen (or a contaminant treated with antibiotics for at least 5 days) in blood or CSF culture within 72 h of birth, regardless of the presence of symptoms. Furthermore, investigators randomly selected 1063 controls from the birth cohort, and each case of EOS was matched with three controls. The most predictive factors for EOS were lower gestational age and higher intrapartum maternal temperature, which accounted for 17 and 58% of the model’s predictive ability, respectively. However, 32% of neonates in the initial cohort with positive blood cultures were asymptomatic, a finding that may potentially overestimate the risk of EOS in the regression model. NSC was updated in 2014 [10] to incorporate into the risk stratification algorithm the results of physical examination (well appearing, equivocal presentation and clinical illness) until the first 24 h of life. For each category of disease, likelihood ratios were identified which, when combined with the risk of sepsis at birth (based on RFs alone), generated a post-test probability of EOS by dividing newborns into 3 risk layers (< 0.65, 0.65–1.54 and > 1.54 cases/1000 live births, respectively). The clinical presentation had effects on the posterior probability for a given EOS risk at birth (likelihood ratio 0.41, 5.0 and 21.2 for well appearing, equivocal and clinical illness, respectively). For each infant the NSC estimated the individual risk of EOS. A posterior risk of EOS < 1, 1–3 and > 3/1000 live births indicated different recommendations (observation, evaluation or antibiotic treatment) [11].

However, in the determination of the final risk, the developers of the model attributed an a priori greater relevance to symptoms, although in absence of strong evidences [12]. Symptoms defined as “equivocal” or “clinical illness” are common in the first hours of life, due to transition to extrauterine life. Developers of NSC introduced safeguards to not discontinue (“strongly consider”) antibiotics in infants with clinical symptoms, even if the posterior probability was below the threshold for treatment (< 3 cases/1000 live births) [11]. This recommendation would increase antibiotic exposure in uninfected infants [12].

### Comparisons between NSC and CM

A systematic review and meta-analysis of published before–after studies regarding NSC [13] included 13 relevant studies and analyzed a total of 175,752 infants. With respect to CM guided strategies, NSC was associated with a reduction in empiric antibiotic use for suspected EOS [relative risk of antibiotic use of 56, 95% C.I. 53–59%]. Rates of missed cases of EOS (defined as an antibiotic initiated after 24 h of life in neonates with culture-positive EOS) were comparable (NSC: 28% vs. CM: 29%, pooled odds ratio, 0.96; 95% C.I., 0.26–3.52; *P* = 0.95). However, NSC does not provide accurate risk estimates with regard to absolute risk of EOS in newborn babies. Estimates might be compromised by technical issues arising with the development and adaptation of the NSC prediction algorithm [12].

### Serial clinical observation (SCO)

An approach entirely based on newborn clinical condition during the first hours of life has recently gained consensus [14, 15]. Asymptomatic neonates undergo a standardized observation (by midwives, nurses and physicians) in the first 48 h of life (Table 2). Laboratory evaluation or empirical antibiotic treatment are only initiated if clinical signs of illness develop [16].

**Table 2:**
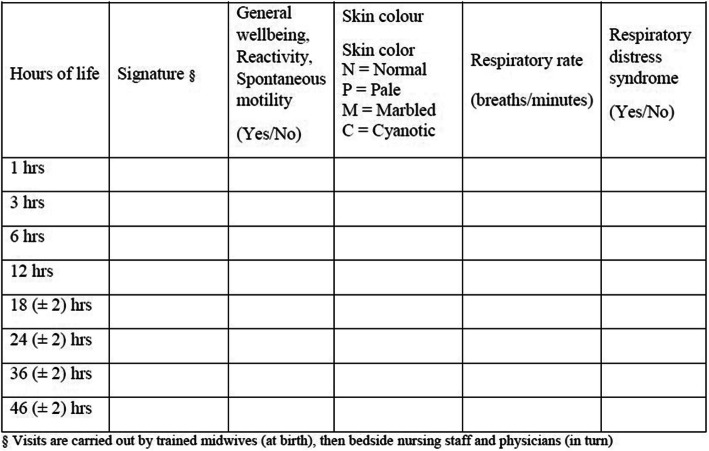
Serial clinical observation approach recommended in Emilia-Romagna (Italy) (modified with permission from ref. [16]). Clinical observation record sheet and timing of visits

A retrospective cohort study at a single Italian centre (20,401 live births from 2005 to 2011) [14] compared a random sample of 500 neonates managed according to CM and 500 neonates managed through SCO. Investigators reported significant reduction of laboratory tests (from 11.6 to 1.6%, *P* < 0.01) and empirical antibiotics (from 2.8 to 0.6%, P < 0.01) following an SCO approach. By reviewing cases of EOS occurring in both periods investigators found no evidence of delayed antibiotic administration or increased risk of neonates to be readmitted for EOS after discharge from hospital. However, the initial symptoms triggering laboratory evaluation or antibiotic therapy were poorly standardized in the original SCO approach, and there was heterogeneity among centers in the timing and ways of performing SCO. This strategy has been recently updated [16, 17].

Minor and major clinical symptoms and criteria most suggestive of EOS during the first hours of life have been recently defined (Table 3).

**Table 3:** Minor and major clinical symptoms and criteria suggesting observation or laboratory evaluation and antibiotic treatment

Minor ‡	Major
Mild respiratory distress (> 60 bpm) without the need of respiratory support	Moderate to severe respiratory distress (requiring respiratory support) § → tachypnoea *plus* increased respiratory effort
Tachycardia > 160 bpm	Hypoxia, reduced SpO2 saturation
Metabolic acidosis (base excess ≤ − 10 mmol/lt)	Reduced skin perfusion, Refill time ≥ 3 “ Signs of shock
Temperature < 36° or > 37.5 < 38 °C	Temperature ≥ 38 °C
	Greyish, pallor or marbling of the skin colour
	Worsening of general wellbeing, apnoea, lethargy, irritability, convulsions

SpO2, Saturation of peripheral oxygen

‡ On the basis of the clinician’s judgment laboratory evaluation can be delayed in the presence of minor, initial, unspecific and non-progressive symptoms during the first 12–24 h of life. Neonates with mild symptoms are re-evaluated at 2-h intervals. The presence of major symptoms, the worsening or persistence (for 12–24 h) of minor symptoms suggest laboratory evaluation and (eventually) empirical antibiotics, but the decision is left to the clinician’s discretion

§ respiratory support includes mechanical ventilation and nasal continuous positive airway pressure. However, it does not necessarily include high flow nasal cannula

SCO recommends observation (without empirical antibiotics) for most neonates with mild, nonspecific symptoms (even in the presence of RFs). Such symptoms are quite different from those suggested by the NSC. Thus, the use of empirical antibiotics is likely to be different. The safety of this updated SCO strategy is under evaluation.

As with the NSC strategy, cases of EOS may be missed with SCO if neonates are selected only on the basis of maternal RFs. A large, multicenter study in Italy reported 48 cases of confirmed group B streptococcus EOS among 265,508 live births managed through an SCO approach; 15 of 48 cases (31.2%) had no RFs for EOS and had symptoms of EOS (three had severe disease) during their hospitalization. Most of them (*n* = 12) were well appearing at birth but subsequently developed symptoms [18].

### Comparisons between NSC and SCO

A retrospective study [19] addressed both safety and empiric antibiotic use for suspected EOS after an SCO or NSC guided approach. Investigators analyzed a cohort of 384 infants of gestational age ≥ 34 weeks undergoing antibiotic therapy in the first 72 h of life. While no cases of EOS would have been missed with both strategies, newborns who would have received antibiotics according to SCO or NSC were 17 and 57% (*p* < 0.001) respectively. However, the selection of neonates for antibiotic treatment was decided a posteriori, after the newborns had already been managed by the clinician.

## Conclusions

Both NSC and SCO may miss cases of EOS, and clinical vigilance for all neonates is essential. Furthermore, unnecessary antibiotics would be reduced by identifying symptoms more predictive of EOS. Studies comparing strategies for managing neonates are recommended.

## Data Availability

Not applicable.

## Abbreviations

EOS: Early-onset sepsis
RFs: Risk factors
NSC: Neonatal sepsis calculator
IAP: Intrapartum antibiotic prophylaxis
SCO: Serial clinical observation
